# The GH/IGF-I Axis and Cognitive Changes across a 4-Year Period in Healthy Adults

**DOI:** 10.5402/2011/249421

**Published:** 2011-04-03

**Authors:** Jan Berend Deijen, Lucia I. Arwert, Madeleine L. Drent

**Affiliations:** ^1^Department of Clinical Neuropsychology, VU University of Amsterdam, van der Boechorst Street 1, 1081 BT Amsterdam, The Netherlands; ^2^Section Endocrinology, Department of Internal Medicine, VU University Medical Center, P.O. Box 7057, 1007 MB Amsterdam, The Netherlands

## Abstract

After the age of 40, the amount of growth hormone in humans decreases. The reduced activity of the GH-IGF axis may play a role in age-related cognitive impairments. In the present study, mood and cognition of 30 healthy subjects (7 males, 23 females, aged 41–76 yr, mean age 60.9 ± 9.0) were examined twice. At baseline, we determined fasting blood levels of GH and IGF-I. Mood and cognitive status were assessed at baseline and after, on the average, 3 years and 9 months of followup. Working memory performance decreased over the years in the low IGF-group (*P* = .007), but not the high IGF-I group. Higher levels of GH were related with a better working memory at the second test (*r* = 0.42, *P* = .01) while higher levels of IGF-I tended to be related with a better working memory (*r* = 0.3, *P* = .06). The results suggest that higher serum levels of GH and IGF-I preserve the quality of working memory functions over the years.

## 1. Introduction

Growth hormone (GH) is secreted from the pituitary gland and stimulates the liver to produce insulin-like growth factor I (IGF-I). After the age of 40 the amount of growth hormone in humans progressively decreases and an increasing age-associated cognitive impairment is seen, as compared to subjects aged between 20 and 30 yrs [1, 2]. As IGF-I levels have been found associated with mood as well as cognitive functioning, the reduced circulating IGF-I levels may play a role in age-related psychological impairments. With respect to mood, adults with growth hormone deficiency (GHD), a pathological state characterized by low IGF-I levels, feel less energetic, are emotionally more labile and experience feelings of social isolation at a significantly higher frequency than controls [3]. With respect to cognitive functions, IGF-I plasma levels of healthy elderly were positively associated with mini mental state examination (MMSE) scores [4]. Similarly, IGF-I levels in elderly healthy men were found to be associated with better performance in tests sensitive to the effects of aging, especially fluid intelligence [5] and verbal fluency and MMSE [6]. With respect to age-related cognitive impairments, higher serum total IGF-I levels in healthy subjects above 55 years were associated with less cognitive decline over the following two years. The Mini-Mental State Examination (MMSE) was used to assess cognitive impairment at baseline and cognitive decline after, on the average, 1.9 year of follow-up [7]. In two studies separate in males and females, cognitive functions of subjects of 65 years and older were related to levels of free IGF-I and IGF-I to IGFBP-3 molar ratio determined from stored blood samples. Cognitive functions were assessed by means of telephone-based tests. Levels of free IGF-I measured in blood samples collected at a mean age of 57 years in men were positively related to global and verbal memory performance on average of 18 years later, and IGF-I levels collected at a mean age of 56 years in women to general cognition on average of 10 years later. These results indicate that higher midlife free IGF-I may be associated with better late-life cognition [8, 9]. Quite recently, low IGF-I levels were found to be associated with cognitive decline in hypertensive elderly subjects aged 65 year and older [10] and with a prolonged latency of the P300 event-related potential, which may predict cognitive decline, in males aged between 30 and 50 years [11].

Increasing knowledge is present on the mechanisms underlying the relationship between the GH-IGF-I axis and brain function. For instance, there is evidence that GH can cross the blood-brain barrier [12], and binding sites for GH and IGF-I are found in the brain in the choroid plexus, hypothalamus, putamen, thalamus, and hippocampus [13, 14]. In these brain areas, which play a role in mood and memory, the number of GH receptors declines with aging [15]. In addition, IGF-I potentiates acetylcholine release from the hippocampus, and aging is associated with a dramatic reduction of IGF-I protein levels and receptor density in this structure [16, 17]. 

IGF-I has important functions in the development and differentiation of the central nervous system [18]. Growth and development of neurons in the dentate gyrus of the hippocampus are modulated by IGF-I. With aging reductions in IGF levels and in neurogenesis are shown. For instance, plasma concentrations of IGF-I, IGF-II and IGFBP3 were found to be reduced in subjects aged 65 to 92 years presenting no malnutrition and no inflammation as compared to healthy controls aged 20 to 65 years [19]. 

As IGF-I levels fall with aging and correlate with cognitive decline, the possible role of IGF-I levels in the development of dementia has been examined. For instance, the relationship between the hypothalamus-pituitary-adrenal function, IGF-I and IGF-binding proteins (IGFBPs) was studied in patients with Alzheimer's disease (AD). Patients with AD had lowered IGF-I and IGFBP-3 levels and higher IGFBP-1 levels compared to controls [20]. IGF-I levels correlated inversely with cognitive impairment. In another study in 49 healthy centenarians (mean age 100.4 year), cognitive functioning was assessed by clinical dementia rating. Centenarians with lower IGF-I levels had higher prevalence of dementia [21].

In a previous study we explored the stimulatory effect of a nutritional supplement on the GH-IGF-I axis and cognition in healthy subjects (aged 40 to 76). Serum GH, IGF-I and cognitive function were assessed at baseline and again after three weeks. Disregarding the effects of treatment, we found that a higher increase in IGF-I across sessions was related to more improvement in working memory and vigor [22].

The objective of the present study was to evaluate whether the GH-IGF-I status determined approximately 4 years earlier in middle-aged and elderly healthy subjects is predictive for their later mood state and/or cognitive functioning. We administered a parallel version of the psychological tests used almost 4 years earlier to the same subjects to evaluate the relationship between previously determined GH/ IGF-I levels and later test performance. We hypothesized that higher IGF-I and/or GH levels are associated with better psychological functions determined four years later.

## 2. Materials and Methods

### 2.1. Subjects

A number of 30 apparently healthy subjects (7 males, 23 females, aged 41 to 76 years, mean age 60.9 ± 9.0) participated in this study. They previously participated in a study on the effect of a nutritional food supplement on GH and IGF-I plasma levels and cognitive function [22]. The total group of subjects participating in the original study consisted of 42 healthy nonobese volunteers, 14 males and 28 females, aged 40 to 76 years. At baseline, blood was collected for the measurement of serum GH and IGF-I levels. At the end of 2005, all participants received a written invitation to take part in a follow-up study, consisting of a parallel version of the psychological tests. From these 42 subjects, 30 responded and underwent a second test administration, separated by about 4 years from the first study. Mean baseline values of the participating subjects were for GH 6.7 nmol/L (SD = 13.2) and for IGF-I 18.2 nmol/L (SD = 6.0). Excluded at baseline were subjects with professional sports participation, vegetarians, alcohol consumers (>3 units/day), dieters, subjects addicted to substance abuse, subjects who used, within 1 month prior to start of the study, hypnotics, antidepressants, or nutritional supplements. The study was conducted according to the Declaration of Helsinki and was approved by the Ethics Committee of the VU University Medical Center. All subjects gave their written informed consent before inclusion in the study. The baseline data collection of this study took place January–February 2002 and the second data collection in October–November 2005.

### 2.2. Tests and Procedure

Subjects were examined individually in a quiet room. The sequence of the tests was the same for all subjects, and the test procedure as a whole took about 45 minutes.

A parallel version of the tests used in the previous study [22], and selected from the Neurobehavioral Evaluation System (NES) [23], was administered by means of a personal computer.

#### 2.2.1. Mood

**The Profile of Mood States (POMS)**

It is a self-administered questionnaire in which the subjects rate themselves with respect to their feelings over the previous 3 days. The shortened Dutch version of 32 items was used, which consists of five subscales: depression, anger, fatigue, vigor, tension and a total mood score (average of all subscales). In the present study, we evaluated fatigue, vigor and total mood. Responses are made by choosing from five response alternatives. Score: The rating (1–5) on each item and the scale to which each item contributes is recorded. With respect to Vigor a higher score corresponds to a better mood state. For the other scales a higher score points to a worse mood state.

#### 2.2.2. Short-Term Memory and Intermediate Memory

**Associate Learning Task**

Nine word pairs consisting of a name and an occupation were displayed on a computer screen at a constant rate of one pair per 3 seconds. After these pairs were presented, the subject was asked to choose one of the nine occupation alternatives. After each answer, the results were given in the following manner: “Yes, John is a grocer,” or “No, Susan is a teacher.” Three trials were given in which the subject has to learn as many paired names and occupations as possible. By means of this recognition procedure short-term verbal learning is measured. Score: the total of correct associations (maximum score: 27).

**Visual Digit Span Task**

A sequence of digits was presented one at a time to the subject. After the whole sequence was presented, the subject was required to enter the sequence on the computer keyboard. Increasingly longer spans of digits were presented, until the subject made two errors at a span length. Score the length of the longest span answered correctly (maximum score: 9).

**Associative Learning Delayed Recognition Task**

To assess intermediate recall, a single recognition trial of the nine names to be matched with one of the nine occupations used in the *associate learning test* was administered at the end of the testing session. Score: the number of correct responses (maximum score: 9).

#### 2.2.3. Working Memory (WM)

**Digit Span Backward**

In the second part of the* visual digit span task* the subject was asked to respond with the order of digits reversed (backward), measuring working memory performance. Score: the length of the longest span answered correctly (maximum score 8).

**WM Composite**

We assessed prefrontal functions by calculating a working memory score composed by adding the score on the first trial of the associate learning task and the score on the digit span backwards task. As these two tasks require the most active manipulation of the material to be remembered a summation of the scores represents a score for working memory capacity. Score: the sum of correct responses of the first trial of the associate learning task and the score on the digit span backwards (maximum score: 17).

### 2.3. Baseline Hormone Assays

Blood samples were taken once, that is at baseline a 5-cc venous blood sample was drawn after an overnight fast for determination of serum GH and IGF-I levels. The blood samples were centrifuged (10 minutes, 3500 r.p.m. at 4°C) and serum was stored at −20°C until analysis.

Serum GH and IGF-I levels were measured using commercially available assays (GH, immunometric assay, Sorin Biomedica, Saluggia Italy; IGF-I, Chemoluminiscentic, Nichols Institute Diagnostics, San Juan Capristrano, USA). The detection limits for GH and IGF-I are 1.0 mU/L (0.5 *μ*g/L) and 0.6 nmol/L, respectively. The intra-assay coefficient of variation (CV) for GH is 4% at serum GH of 4 mU/L. The interassay coefficient of variation for GH is 9% at serum GH of 2.5 mU/L and 8% at serum GH of 8.6 mU/L. For IGF-I the intra-assay coefficient of variation is 3% at serum IGF-I of 20 nmol/L and the inter assay coefficient of variation is 6% at serum IGF-I of 33 nmol/L and 8% at serum IGF-I of 7 nmol/L.

### 2.4. Data Analysis

Data were analysed by means of ANOVA with groups (low/high GH/IGF-I) as independent factor and session as repeated measurements factor (original baseline and present study). Subjects with GH values above the median GH (median GH = 2.25 nmol/L) were assigned to the high GH group (*n* = 15) and those with values below the median to the low GH group (*n* = 15). Similarly, a distinction was made between a group with IGF-I values above (*n* = 15) and a group with values below the median (*n* = 15; median IGF-I = 17.5 nmol/L). In case an interaction was found between group and session, paired *t*-tests (one-tailed) were performed. In addition, we calculated the bivariate Pearson correlation coefficient between GH and IGF-I levels and the partial correlation coefficient between GH/IGF-I values and test scores at the second session controlling for the baseline test scores. 

Significance level was defined as *P* ≤ .05. Tests were one tailed, based on the assumption that higher GH/IGF-I values would be associated with better test scores at the second session relative to baseline. Data were analysed using the SPSS version 14 software package (SPSS Inc., Chicago, USA).

## 3. Results

The serum GH and IGF-I levels of 30 subjects out of the original group of 42 subjects could be related to scores on the psychological tests. Baseline data were similar for the 30 volunteers and 12 nonparticipating subjects, except for GH level, nonparticipating subjects having lower GH (but not IGF-I) levels (*P* = .03). 

With regard to the GH-IGF-I axis, the Pearson correlation between GH and IGF-I values was significant (*r* = 0.43, *P* = .009). With respect to the GH groups, the mean GH value in the low group was 0.8 mU/L (SD = 0.6) and in the high group 12.5 mU/L (SD = 16.9). In addition, the mean IGF-I value in the low IGF-I group was 13.8 nmol/L (SD = 2.8) and in the high IGF-I group 22.5 nmol/L (SD = 5.05). ANOVA indicated a significant main effect for session for digit span backward (baseline, mean = 5.2, SD = 1.7; session 2, mean = 4.47, SD = 1.3; *F* (1, 28) = 8.5, *P* = .007, *η* ^2^ = 0.23) and WM composite (Baseline, mean = 9.23, SD = 2.5; session 2, mean = 8.17, SD = 2.27; *F *(1,28) = 4.8, *P* = .037, *η* ^2^ = 0.15. This means that working memory performance was decreased in the total group of subjects during the 4-year interval. Mean values of mood and memory scores are presented in Table 1. 

**Figure 1:**
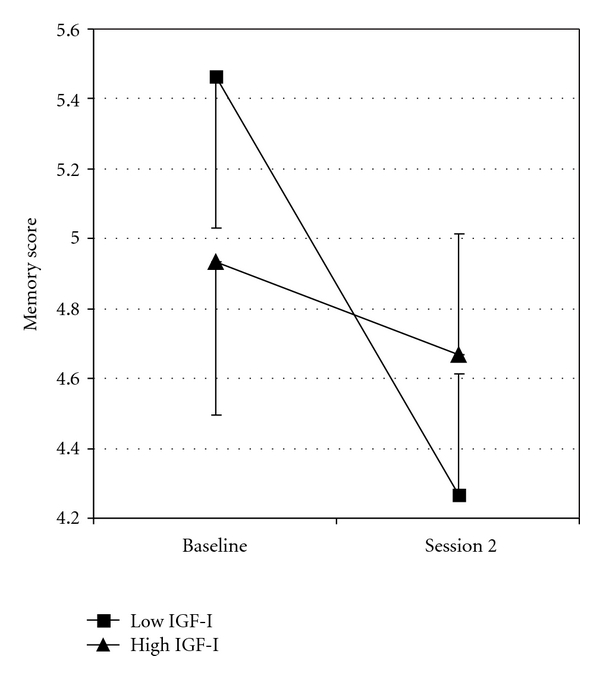
Mean scores (±SE) on digit span backward at baseline and session 2 for the low (*n* = 15; mean IGF-I = 13.8 nmol/L) and high (*n* = 15; mean IGF-I = 22.5 nmol/L) IGF-I groups.

With respect to the POMS, an interaction between GH or IGF-I group and session was not found, indicating no differential effect of GH/IGF-I group on mood parameters. Regarding the memory tests, there was no interaction between GH group and session. However, a tendency for an interaction between IGF-I group and session was found for digit span backwards and WM composite (*F *(1,28) = 3.4, *P* = .07, *η* ^2^ = 0.11, and *F *(1,28) = 3.2, *P* = .086, *η* ^2^ = 0.1, resp.). Paired *t*-tests indicated a significant decrease in digit span backward and WM composite in the low IGF-I group (*t*(14) = 2.08, *P* = .007, and *t*(14) = 2.6, *P* = .02, resp.) whereas no significant effect was found in the high IGF-I group. This result indicates that working memory performance in the low IGF-group decreased across the years while the working memory in the high IGF-I group was not significantly decreased relative to baseline (Figure 1). 

In addition to the ANOVA with repeated measurements, we calculated the partial correlation coefficient between the baseline values of at one hand GH and IGF-I and on the other hand the psychological test scores at the second test session. The baseline test scores were controlled for. There were no significant correlations between GH or IGF-I with mood state parameters. However, GH correlated significantly with the scores on the first trial of associate learning (*r* = 0.39,  *P* = .02) while IGF-I values tended to correlate with the scores on this test (*r* = 0.25, *P* = .09). With respect to WM composite, GH significantly correlated with these scores (*r* = 0.42, *P* = .01) and IGF-I tended to correlate (*r* = 0.3, *P* = .06) suggesting that higher GH and IGF-I values are related with a better working memory at the second test session.

## 4. Discussion

In the present study, we examined whether higher concentrations of GH and/or IGF-I levels in healthy adults above 40 years are indicative of their psychological functions about 4 years later. Contrary to our previously observed associations between IGF-I levels and mood parameters in healthy subjects [22], we found no association between IGF-I concentration and mood status. The results of the analyses of variance indicate that the concentration of IGF-I was related to memory performance determined 4 years later. The group with higher IGF-I levels showed a relatively better memory performance at the second test session. In contrast, no differences between the low and high GH groups were seen with respect to psychological test scores. The correlation analyses indicated significant associations between GH values and memory and a trend towards significant associations between IGF-I values and memory. As GH levels were only determined in one daily sample, it was surprising to observe significant associations between GH and memory parameters. A single determination of the GH concentration is assumed not to be very reliable because GH is secreted in a pulsatile fashion, with 2/3 of the daily secretion taking place at night. However, although in the present study the correlation coefficient between concentrations of GH and IGF-I levels was not very substantial, the levels were significantly associated. Thus, a single determination of GH may yet be reliable enough to reveal relationships between the GH/IGF-I axis and psychological parameters. Indeed, it has been shown that, because of homeostatic control mechanisms within the GH-IGF-I axis, the day to day mean mass of GH secretion per burst and the serial orderliness of the GH release in healthy persons is strongly preserved across a wide span of ages and body-compositions [24]. For instance, the intraindividual mean mass of GH secretion per burst has been found to be highly conserved across sessions (*r* = 0.93) and consecutive days (*r* = 0.92) in healthy men (age range, 29–77 yr) from whom every 10 min blood samples were drawn during 48 hours. As we did not observe differences between the high and low GH groups on psychological parameters but yet found that GH and cognitive functions were correlated, it may well be true that the interindividual and intraindividual variability in GH levels results in a higher sensitivity to observe associations between the GH/IGF-axis and memory functions than is the case for IGF-I. Thus, the more stable IGF-I marker for GH status may be more appropriate for discerning cognitive differences between IGF-I groups, but less sensitive for detecting interindividual associations between GH/IGF-I axis and cognitive parameters. 

The present results indicate that there is a relationship between GH and IGF-I levels and cognitive performance in healthy adults across the years. Higher levels of GH and IGF-I seem to be related to a better memory performance in the consecutive years. Regrettably, because of practical constraints, we were not able to take blood samples for determination of serum GH and IGF-I at the second test session. It may thus well be true that intermediating factors account for the observed relationship between GH/IGF-I and memory. One such factor may be lifestyle. For example, food or sport habits associated with the original baseline GH and IGF-I serum levels may explain the better memory performance at the second test session. Although professional sports participants and users of nutritional supplements were excluded at baseline, it cannot be excluded that subjects differed with respect to healthy life style habits. A healthier life style may improve mental functions directly or indirectly by increasing GH/IGF-I levels.

The results of the present study suggest a relation between the GH/IGF-I axis and working memory. The digit span backward task used in the present study may be assumed to be quite powerful, because remembering in the reverse order increases task load substantially. Indeed, the high and low IGF-I groups were distinguishable on this task, the high IGF-I group performing better. Thus, especially the performance on a working memory task with high task load may differentiate groups. The effects of the GH/IGF axis on memory may be attributed to neurophysiological mechanisms associated with the regenerative action of IGF-I, but also GH, in the adult CNS or be due to an increase of locally produced IGF-I within the brain [25]. Specifically, these underlying mechanisms may be related to the presence of IGF-I receptors in the frontal cortex and to neuroprotective effects of IGF-I with respect to cholinergic neurons in the forebrain [26]. In addition, as is stated in the Introduction, IGF potentiates acetylcholine release from the hippocampus and modulates growth and development of neurons in the dentate gyrus of the hippocampus [16–18]. The involvement of the GH/IGF-I axis in the activity of the hippocampus and prefrontal cortex is associated with the N-methyl-D-aspartate (NMDA) receptor. GH substitution in GH-deficient patients has been found to increase CSF levels of aspartate [12], an excitatory amino acid which is a ligand for the N-methyl-D-aspartate (NMDA) receptor. Activation of the NMDA receptor contributes to long-term potentiation of synaptic efficacy in the hippocampus, which is considered to be an inherent mechanism of memory consolidation in the mammalian brain [27]. Moreover, it has been shown that activation of NMDA receptors in the basal forebrain modifies attentional functions [28]. 

A former fMRI study showed that GH-deficient patients had a higher than normal activity in parietal cortex and prefrontal cortex during the performance of a working memory task, which may be due to compensatory recruitment of these brain regions [29]. These findings indicate that the GH/IGF-I axis contributes to prefrontal functioning and may thus mediate and preserve memory functions.

Overall, we conclude that circulating GH, and IGF-I levels may influence cognitive functions, higher serum levels of GH and IGF-I preserving the quality of working memory functions over the years.

## Figures and Tables

**Table 1:** Mean mood and memory performance scores (±SD) for the low (*n* = 15; mean IGF-I = 13.8 nmol/L) and high (*n* = 15; mean IGF-I = 22.5 nmol/L) IGF-I groups. Higher scores for fatigue, mood total, and symbol-digit correspond to worse performance. Higher scores for the other tests correspond to better performance.

	Low IGF-I		High IGF-I	
	Baseline	Session 2	Baseline	Session 2
Vigor	3.25 ± 0.8	3.48 ± 0.7	3.39 ± 0.91	3.23 ± 1.21
Fatigue	2.73 ± 0.65	2.52 ± 0.7	2.84 ± 0.95	2.77 ± 1.21
Mood total	1.51 ± 0.31	1.55 ± 0.3	1.64 ± 0.41	1.64 ± 0.62
Associate learning trial 1	4.33 ± 2.09	3.6 ± 1.96	3.37 ± 1.7	3.80 ± 2.21
Associate learning trial 2	5.0 ± 1.81	4.67 ± 2.44	4.8 ± 1.97	4.53 ± 2.1
Associate learning trial 3	6.2 ± 2.18	6.13 ± 1.64	5.2 ± 2.30	5.4 ± 1.80
Associate recall	6.4 ± 2.2	5.6 ± 2.77	5.13 ± 2.10	5.47 ± 2.39
Digit span forward	6.13 ± 1.64	6.0 ± 1.0	6.0 ± 1.56	5.53 ± 1.64
Digit span backward	5.47 ± 1.55	4.27 ± 1.22	4.93 ± 1.83	4.67 ± 1.44
Symbol-digit (error)	2.47 ± 5.54	1.6 ± 2.67	3.36 ± 4.74	1.45 ± 1.69
WM composite	9.8 ± 2.48	7.87 ± 2.10	8.67 ± 3.13	8.47 ± 2.47
